# Effect of Some Psychoactive Drugs Used as ‘Legal Highs’ on Brain Neurotransmitters

**DOI:** 10.1007/s12640-015-9569-1

**Published:** 2015-10-26

**Authors:** Krystyna Gołembiowska, Alexandra Jurczak, Katarzyna Kamińska, Karolina Noworyta-Sokołowska, Anna Górska

**Affiliations:** Department of Pharmacology, Institute of Pharmacology, Polish Academy of Sciences, 12 Smętna, 31-343 Kraków, Poland

**Keywords:** Mephedrone, PMA, PMMA, MDMA, DA, 5-HT, Rat brain

## Abstract

New psychoactive “designer drugs” are synthetic compounds developed to provide similar effects to illicit drugs of abuse, but not subjected to legal control. The rapidly changing legal status of novel psychoactive drugs triggers the development of new compounds, analogs of well-known amphetamine or mescaline. New designer drugs used as substitutes in ecstasy pills are the least investigated and can cause life-threatening effects on users. The aim of our research was to examine the effects of acute administration of 4-methoxyamphetamine (PMA, 5 and 10 mg/kg), 4-methoxy-*N*-methylamphetamine (PMMA, 5 and 10 mg/kg), and mephedrone (MEPH, 5, 10 and 20 mg/kg) on extracellular and tissue level of dopamine (DA), 5-hydroxytryptamine (5-HT) and their metabolites in rat brain, by microdialysis method in freely moving animals and HPLC. Similarly to 3,4-methylenedioxymethamphetamine (MDMA, 5 and 10 mg/kg) PMA, PMMA and MEPH enhanced the release of DA and 5-HT in rat striatum, nucleus accumbens, and frontal cortex. DA tissue content was increased by MEPH and PMMA in striatum, by MEPH, PMA, and PMMA in nucleus accumbens, and by PMA in frontal cortex. Instead, cortical DA level was decreased by MEPH and PMMA. MEPH did not influence 5-HT tissue level in striatum and nucleus accumbens, but decreased its level in frontal cortex. PMMA increased 5-HT content in striatum, while PMA enhanced it in nucleus accumbens and frontal cortex. Observed changes in brain monoamines and their metabolites by new psychoactive drugs suggest that these drugs may be capable of development of dependence. Further experiments are needed to fully investigate the neurotoxic and abuse potential of those drugs.

## Introduction

According to recent EMCDDA report, over the past 5 years, there has been an unprecedented increase in the number, type, and availability of new psychoactive substances in Europe, which replace their illegal counterparts and are easily obtainable on the Internet. Continuing this trend, during 2014, a total of 101 new substances were reported for the first time to the EU Early Warning System (EMCDA 2014). It is likely that the growth of the market in new psychoactive substances will continue to pose a range of challenges for public health and drug policy over the next few years, especially due to their unknown pharmacologic and neurotoxic effects.

4-Methoxyamphetamine (PMA) and 4-methoxy-*N*-methylamphetamine (PMMA) are methoxylated phenylethylamine derivatives first encountered on the illegal market in 1970s. Because of their similarity to 3,4-methylenedioxymethamphetamine (MDMA) and replacing it in “ecstasy” tablets, they are widespread among young people and consumed without any safe testing (Daws et al. 2000). They are toxic at lower doses than MDMA (Johansen et al. 2003; Lurie et al. 2012) and evoke a delayed effect after ingestion (Felgate et al. 1998), which can lead to an increased intake. Clinical symptoms specific to PMA and PMMA poisoning include life-threatening hyperthermia, tachycardia, rhabdomyolysis, breathing problems, and acute renal failure (Caldicott et al. 2003; Dal Cason 2001; Johansen et al. 2003). The effect evoked by PMA and PMMA is mediated by their interaction with either 5-HT transporter (SERT) or dopamine transporter (DAT) to release those neurotransmitters and inhibit their uptake from presynaptic sites (Callaghan et al. 2005; Daws et al. 2000). However, long-term effects of their action are poorly understood. Studies in rodents suggest that PMA and PMMA are capable of producing acute serotonergic as well as dopaminergic neurotoxicity, although they are less potent than MDMA, which produces dose-dependent selective degeneration of 5-HT terminals in just 7 days after administration (Battaglia et al. 1987; Gough et al. 2002; Gudelsky and Yamamoto 2003; Molliver et al. 1990; Steele et al. 1992).

Additionally, in drug discrimination studies, PMA and PMMA show similarity to MDMA, which suggests their addictive potential (Dukat et al. 2002; Glennon et al. 1997, 2007; Young et al. 1999). These studies raise concern over the illicit use of amphetamine derivatives in combination as “UFO pills.” Recently, the effect of acute doses of PMA and PMMA was investigated and has been proved to cause a release of DA and 5-HT in rat striatum and hippocampus (Matsumoto et al. 2014). A study by Callaghan and co-workers showed that repeated administration of PMA resulted in reductions in hippocampal SERT binding, synaptosomal 5-HT uptake, as well as 5-HIAA content in the cortex, but it remained without effect on 5-HT content (Callaghan et al. 2006). These data suggest that PMA has severe long-term implications for altering the 5-HT neurotransmission, but degeneration of serotonergic fibers is not clear.

Mephedrone (MEPH, 4-methylmethcathinone) is a synthetic derivative of cathinone that can be extracted from the leaves of khat plant (Catha edulis). Structurally, it is a substituted phenethylamine, and as beta-keto-analog of PMMA has powerful psychostimulant and entactogenic properties (Schifano et al. 2011). There is no much evidence on neurotoxicity of mephedrone; however, its psychomimetic effects are comparable to amphetamines since euphoria, elevated mood, and sexual stimulation were reported (Kehr et al. 2011). However, excessive intake leads to acute intoxication characterized by elevated heart rate, increased body temperature, chest pain, tremor, and convulsions (Wood and Dargan 2012; Zawilska 2014; Zawilska and Wojcieszak 2013). What is more, mephedrone has comparable abuse potential to cocaine or ecstasy, triggering repetitive and uncontrolled drug intake (McElrath and O’Neill 2011). There is limited information regarding pharmacodynamic mechanism of mephedrone action, although its profile of action is likely to be comparable with MDMA (Zawilska 2014; Zawilska and Wojcieszak 2013). Initial reports suggest that mephedrone interacts with plasma membrane monoamine transporters (Baumann et al. 2012; Hadlock et al. 2011), blocks the neurotransmitter reuptake (Simmler et al. 2014), and stimulates their release to the synaptic cleft (Kehr et al. 2011). Recent studies in animal models have shown that repeated mephedrone injections in a pattern used to mimic psychostimulant “binge” treatment cause rapid decrease in striatal DA and hippocampal 5-HT transporter function, which suggests its neurotoxic nature (Martínez-Clemente et al. 2014). Mephedrone as well as amphetamine analogs’ neurotoxicity could be mediated by increased cytosolic pool of both DA and 5-HT, which under conditions of vesicular transporter (VMAT2) blockade induce the generation of reactive oxygen species (ROS) as well as dihydroxybitryptamine toxic products, by means of neuronal monoamine metabolism (Colado et al. 2001; Sprague and Nichols 1995; Yamamoto et al. 1995; Wrona and Dryhurst 2001). Additionally, pharmacokinetic studies in rodents and humans have shown that mephedrone is metabolized in a similar pattern to ring-substituted amphetamines, suggesting that bioactive metabolites could be formed in vivo (Meyer et al. 2010).

The purpose of our study was to further complement, by use of in vivo microdialysis, the fundamental knowledge on the effects of selected psychostimulants on the central nervous system. We investigated the changes in DA and 5-HT release in striatum, nucleus accumbens septi, and frontal cortex, following single administration of PMA, PMMA, and MEPH, in the adult rat model. Additionally, we assayed the tissue content of monoamines and their metabolites after acute pretreatment with those psychostimulants. The effect of MDMA on DA and 5-HT release in various brain regions was also assessed for comparison.

## Materials and Methods

### Animals

The study was carried out on male Wistar-Han rats (Charles Rivers, Sulzfeld, Germany) weighing 280–300 g. The animals were housed in temperature- and humidity-controlled rooms under a 12-h light/12-h dark cycle, and had free access to standard laboratory food and tap water. The experiments were conducted in accordance with the European Union guidelines regarding the care and use of laboratory animals (Council Directive 86/609/EEC of November 24, 1986) and were approved by the II Local Bioethics Commission (Institute of Pharmacology, PAS, Kraków, Poland).

### Drugs and Reagents

**Fig. 1:**
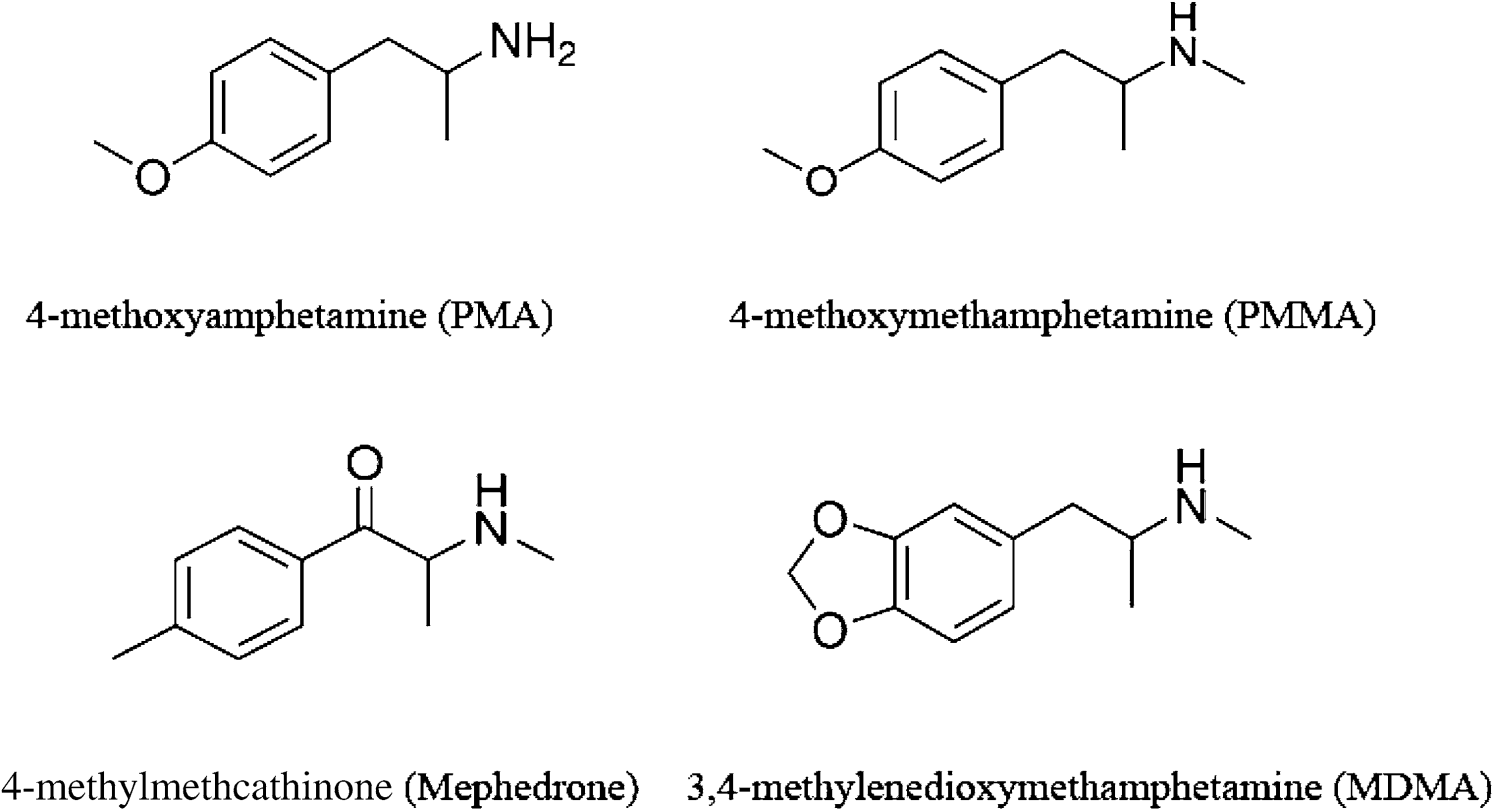
Chemical structure of drugs used in this study

3,4-Methylenedioxymethamphetamine (MDMA), 4-methoxyamphetamine (PMA), and 4-methoxy-*N*-methylamphetamine (PMMA) were purchased from Toronto Research Chemicals Inc. (Canada). (±)-4-Methylmethcathinone (mephedrone) was purchased from Chemwatch (Australia). The chemicals used for HPLC were obtained from Merck (Warsaw, Poland), while ketamine hydrochloride and xylazine hydrochloride were purchased from Biowet (Puławy, Poland). Chemical structure of drugs is shown in Fig. 1.

### Brain Microdialysis

Animals were anesthetized with ketamine (75 mg/kg) and xylazine (10 mg/kg), and vertical microdialysis probes were implanted into the striatum, nucleus accumbens, and frontal cortex, respectively, using the following coordinates: AP +1.8, L +3.0, V −7.0; AP +1.6, L +1.1, V −8.0; AP +2.8, L +0.8, V −6.0 from the dura (Paxinos and Watson 1999). On the next day, probe inlets were connected to a syringe pump (BAS, IN, USA) which delivered artificial spinal fluid (aCSF) composed of [mM]: NaCl 147, KCl 2.7, MgCl_2_ 1.0, CaCl_2_ 1.2; pH 7.4 at a flow rate of 2 µl/min. After 2 h of the washout period, three basal dialysate samples were collected every 20 min; then animals were injected intraperitoneally with appropriate drugs in doses indicated in figure captions and fraction collection continued for 180 min. At the end of the experiment, the rats were sacrificed and their brains were histologically examined to validate the probe placement.

### Analytical Procedure

DA, 5-HT, 3,4-dihydroxyphenylacetic acid (DOPAC), homovanillic acid (HVA), and 5-hydroxyindoleacetic acid (5-HIAA) were analyzed by high-performance liquid chromatography (HPLC) with coulochemical detection. Chromatography was performed using an Ultimate 3000 System (Dionex, USA), coulochemical detector Coulochem III (model 5300, ESA, USA) with 5020 guard cell, 5014B microdialysis cell, and Hypersil Gold C18 analytical column (3 μm, 3 × 100 mm). The mobile phase was composed of 0.1 M potassium phosphate buffer adjusted to pH 3.6, 0.5 mM Na_2_ EDTA, 16 mg/L 1-octanesulfonic acid sodium salt, and 2 % methanol. The flow rate during analysis was set at 0.7 ml/min. The applied potential of a guard cell was 600 mV, while those of microdialysis cells were *E*1 = −50 mV and *E*2 = 300 mV with a sensitivity set at 50 nA/V. The chromatographic data were processed by Chromeleon v. 6.80 (Dionex, USA) software run on a PC computer.

### The Tissue Content of DA, 5-HT, and Their Metabolites

Animals were sacrificed by decapitation 3 h after intraperitoneal drugs administration. Brains were separated and several brain regions (striatum, nucleus accumbens septi, frontal cortex) were dissected in anatomical borders. The tissue levels of DA, 5-HT, DOPAC, HVA, and 5-HIAA were measured using a high-performance liquid chromatography (HPLC) with electrochemical detection. Briefly, tissue samples of brain structures were homogenized in ice-cold 0.1 M HClO_4_ and were centrifuged at 10,000*g* for 10 min at 4 °C. The supernatant (3–5 µL) was injected into the HPLC system. The chromatography system consisted of an LC-4C amperometric detector with a cross-flow detector cell (BAS, IN, USA), a Ultimate 3000 pump (Thermo Scientific, USA), and a Hypersil Gold analytical column (3 μm, 100 × 3 mm, Thermo Scientific, USA). The mobile phase consisted of 0.1 M KH_2_PO_4_, 0.5 mM Na_2_EDTA, 80 mg/L sodium 1-octanesulfonate, and a 4 % methanol, adjusted to pH 3.7 with an 85 % H_3_PO_4_. The flow rate was 1 mL/min. The potential of a 3-mm glassy carbon electrode was set at 0.7 V with sensitivity of 5 nA/V. The temperature of the column was maintained at 30 °C. The Chromax 2007 program (Pol-Lab, Warszawa, Poland) was used for data collection and analysis.

### Data Analysis

All obtained data were presented as a percent of the basal level assumed as 100 %. The statistical significance was calculated using a repeated measures ANOVA or where appropriate a one-way ANOVA, followed by Tukey’s post hoc test. The results were considered statistically significant when *P* < 0.05.

## Results

### DA and 5-HT Release in the Rat Striatum, Nucleus Accumbens, and Frontal Cortex After Administration of PMA

**Fig. 2:**
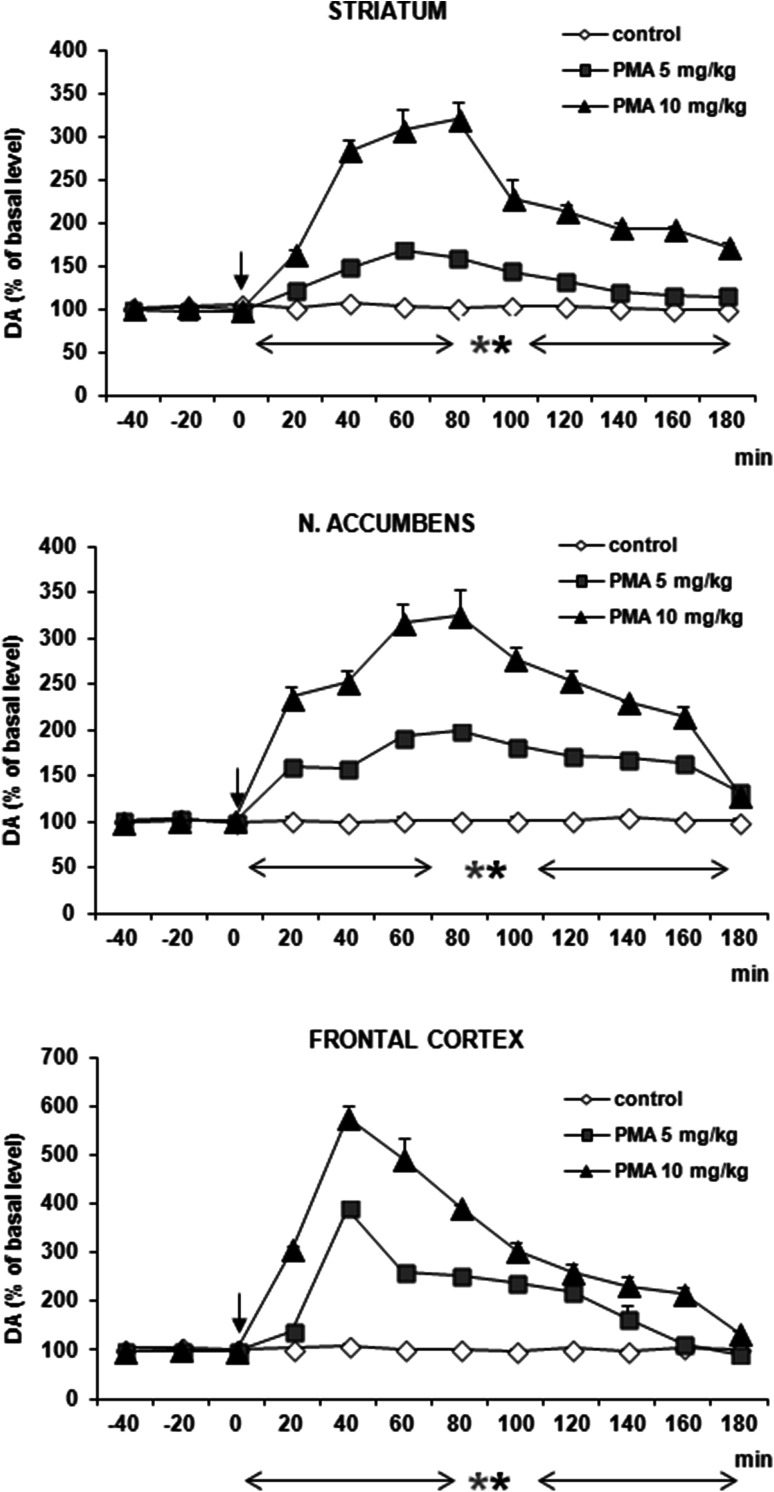
Effect of PMA on DA release in rat striatum, nucleus accumbens, and frontal cortex. Data are mean ± SEM (*n* = 4–5). Drug administration is indicated with an arrow. Basal extracellular level of DA (pg/10 µl) was 18.9 ± 0.8 (striatum); 0.46 ± 0.05 (n. accumbens); and 1.20 ± 0.14 (frontal cortex). *P < 0.01 in comparison to control group (repeated measures ANOVA and Tukey’s post hoc test)

PMA in doses of 5 and 10 mg/kg significantly increased DA release by ca. 300, 550, and 300 % of basal level at 40–80 min after administration in striatum, frontal cortex, and nucleus accumbens, respectively (Fig. 2). Repeated measures ANOVA of these data showed a statistically significant effect of treatment groups [*F*_2,11_ = 121, *P* = 0 in striatum; *F*_2,11_ = 342, *P* = 0 in frontal cortex; *F*_2,11_ = 1329, *P* = 0 in nucleus accumbens], sampling period [*F*_8,88_ = 32, *P* = 0 in striatum; *F*_8,88_ = 95, *P* = 0 in frontal cortex; *F*_8,88_ = 34, *P* = 0 in nucleus accumbens], and the interaction between treatment groups and sampling period [*F*_16,88_ = 13, *P* = 0 in striatum; *F*_16,88_ = 28, *P* = 0 in frontal cortex; *F*_16,88_ = 16, *P* = 0 in nucleus accumbens].

**Fig. 3:**
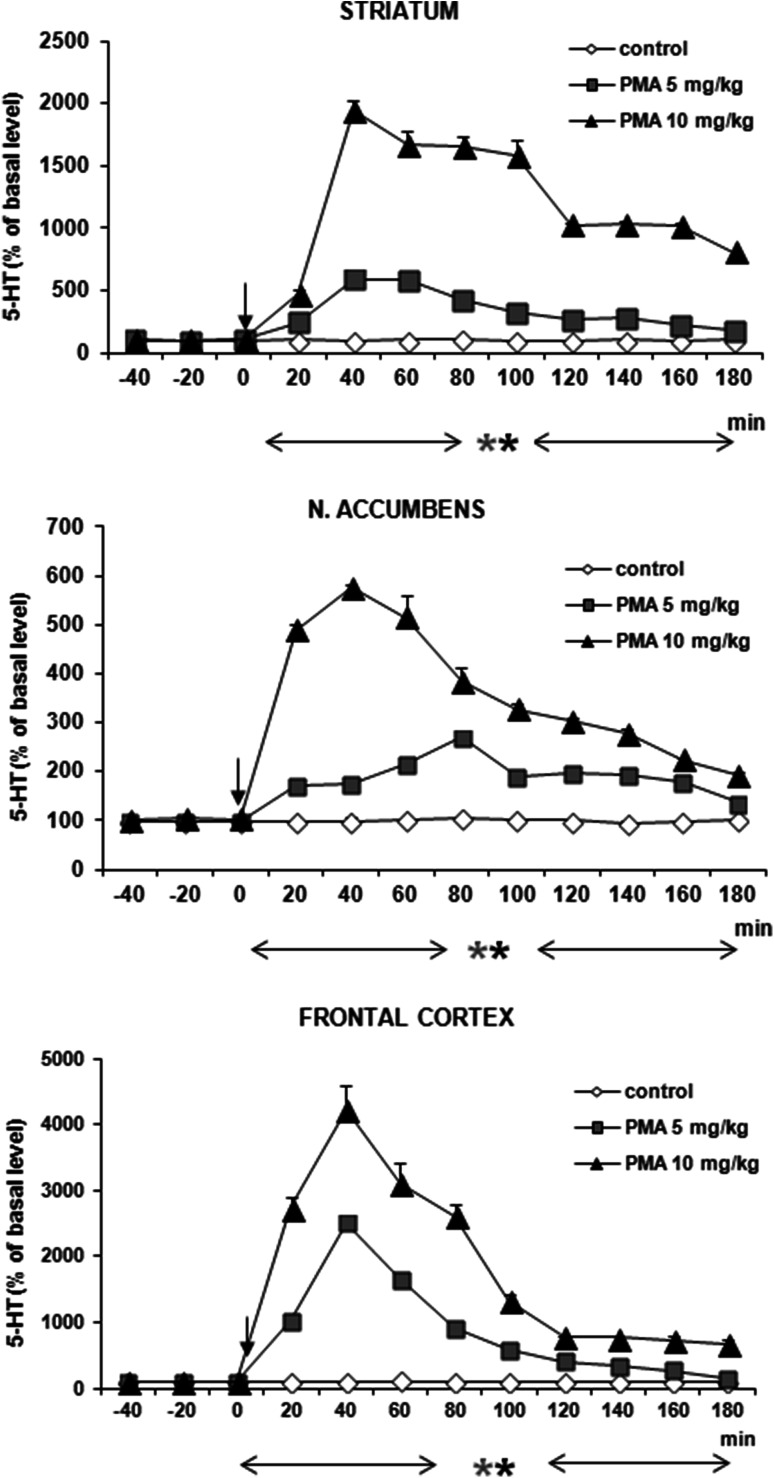
Effect of PMA on 5-HT release in rat striatum, nucleus accumbens, and frontal cortex. Data are mean ± SEM (*n* = 4–5). Drug administration is indicated with an arrow. Basal extracellular level of 5-HT (pg/10 μl) was 0.56 ± 0.04 (striatum); 0.14 ± 0.02 (n. accumbens); and 0.43 ± 0.06 (frontal cortex). *P < 0.01 in comparison to control group (repeated measures ANOVA and Tukey’s post hoc test)

5-HT release was increased by PMA at both doses (5 and 10 mg/kg) in striatum, frontal cortex, and nucleus accumbens by ca. 2000, 4000, and 500 % of basal level, respectively, at 40 min after administration (Fig. 3). Repeated measures ANOVA of these data showed a statistically significant effect of treatment groups [*F*_2,11_ = 577, *P* = 0 in striatum; *F*_2,11_ = 235, *P* = 0 in frontal cortex; *F*_2,11_ = 1883, *P* = 0 in nucleus accumbens], sampling period [*F*_8,88_ = 48, *P* = 0 in striatum; *F*_8,88_ = 102, *P* = 0 in frontal cortex; *F*_8,88_ = 72, *P* = 0 in nucleus accumbens], and the interaction between treatment groups and sampling period [*F*_16,88_ = 25, *P* = 0 in striatum; *F*_16,88_ = 31, *P* = 0 in frontal cortex; *F*_16,88_ = 61, *P* = 0 in nucleus accumbens].

### DA and 5-HT Release in the Rat Striatum, Nucleus Accumbens, and Frontal Cortex After Administration of PMMA

**Fig. 4:**
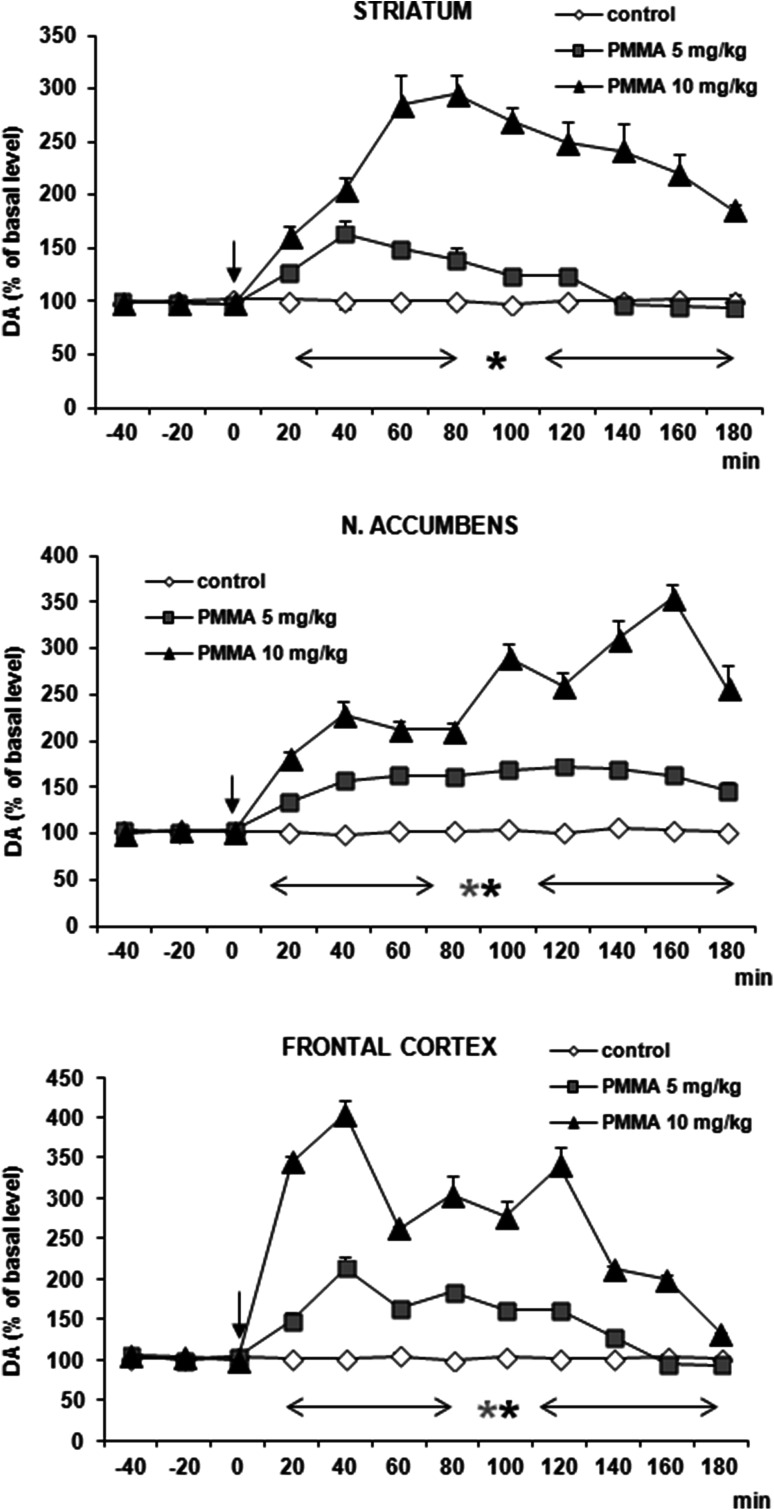
Effect of PMMA on DA release in rat striatum, nucleus accumbens, and frontal cortex. Data are mean ± SEM (*n* = 4–5). Drug administration is indicated with an arrow. Basal extracellular level of DA (pg/10 µl) was 16.3 ± 1.2 (striatum); 0.81 ± 0.07 (n. accumbens); and 0.60 ± 0.05 (frontal cortex). *P < 0.01 in comparison to control group (repeated measures ANOVA and Tukey’s post hoc test)

**Fig. 5:**
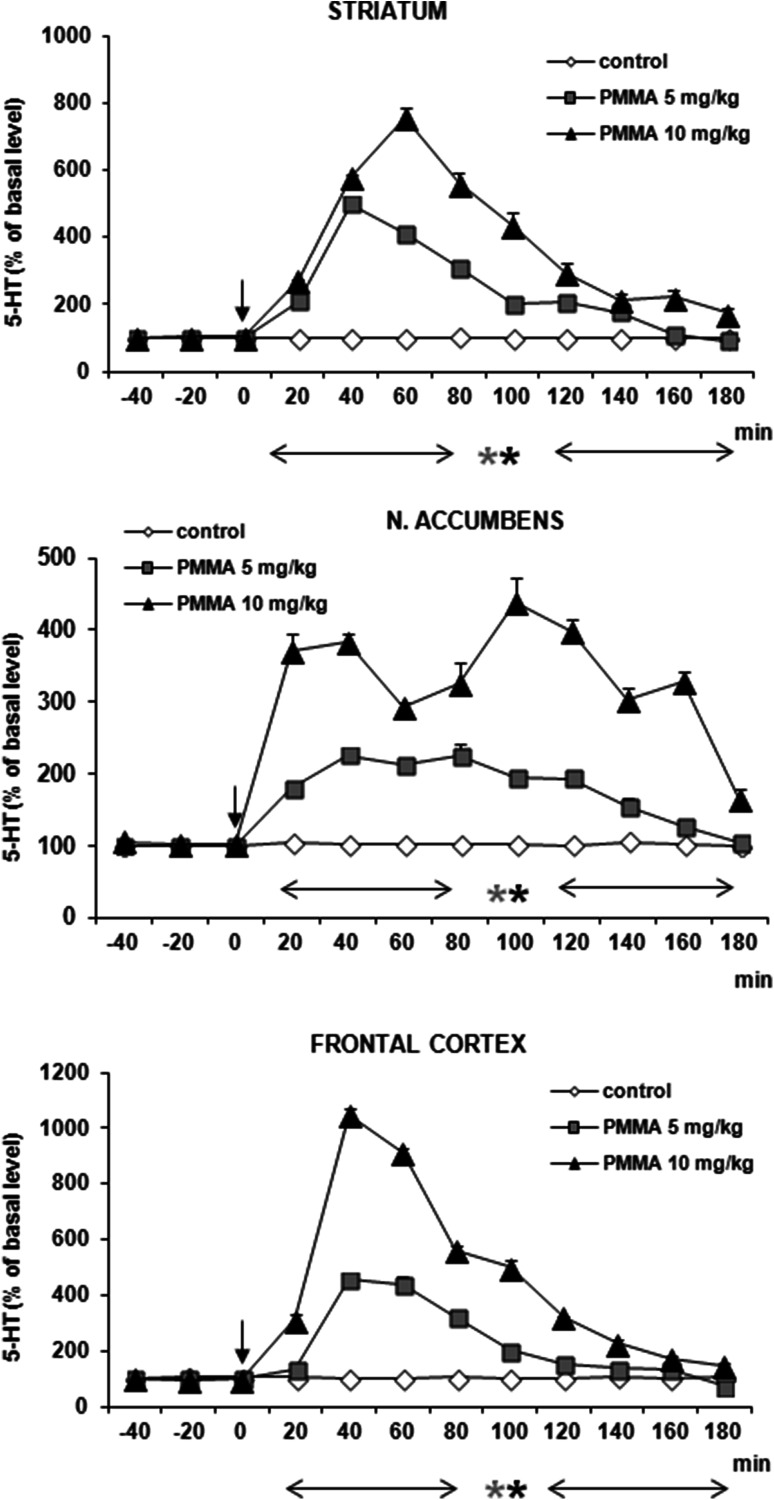
Effect of PMMA on 5-HT release in rat striatum, nucleus accumbens and frontal cortex. Data are mean ± SEM (*n* = 4–5). Drug administration is indicated with an arrow. Basal extracellular level of 5-HT (pg/10 µl) was 0.40 ± 0.9 (striatum); 0.22 ± 0.03 (n. accumbens); and 0.25 ± 0.04 (frontal cortex). *P < 0.01 in comparison to control group (repeated measures ANOVA and Tukey’s post hoc test)

PMMA significantly increased DA release in both doses (5 and 10 mg/kg) in frontal cortex and nucleus accumbens and at the higher dose in striatum only (Fig. 4). DA release was enhanced by ca. 300–400 % of basal level in all studied brain regions at 60–80 min after administration. 5-HT release was increased by PMMA in both doses (5 and 10 mg/kg) by ca. 700, 1000, and 400 % of basal level at 40–100 min after administration in striatum, frontal cortex, and nucleus accumbens, respectively (Fig. 5). Repeated measures ANOVA of these data showed a statistically significant effect of treatment groups [*F*_2,11_ = 158, *P* = 0 in striatum; *F*_2,11_ = 5092, *P* = 0 in frontal cortex; *F*_2,11_ = 393, *P* = 0 in nucleus accumbens], sampling period [*F*_8,88_ = 190, *P* = 0 in striatum; *F*_8,88_ = 691, *P* = 0 in frontal cortex; *F*_8,88_ = 48, *P* = 0 in nucleus accumbens], and the interaction between treatment groups and sampling period [*F*_16,88_ = 60, *P* = 0 in striatum; *F*_16,88_ = 255, *P* = 0 in frontal cortex; *F*_16,88_ = 23, *P* = 0 in nucleus accumbens].

### DA and 5-HT Release in the Rat Striatum, Nucleus Accumbens, and Frontal Cortex After Administration of Mephedrone

**Fig. 6:**
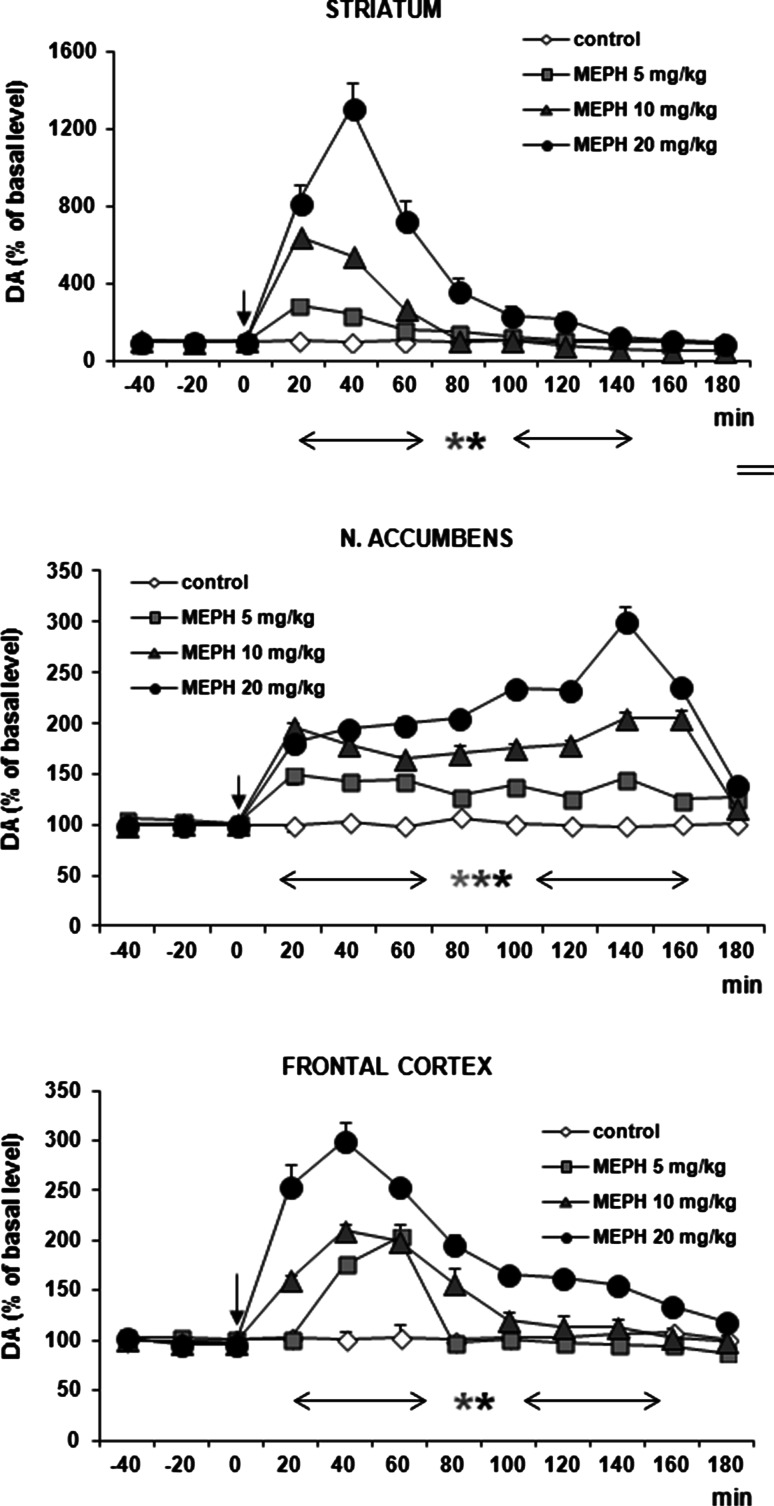
Effect of mephedrone (MEPH) on DA release in rat striatum, nucleus accumbens, and frontal cortex. Data are mean ± SEM (*n* = 4–5). Drug administration is indicated with an arrow. Basal extracellular level of DA (pg/10 μl) was 18.7 ± 1.6 (striatum); 0.67 ± 0.07 (n. accumbens); and 0.57 ± 0.06 (frontal cortex). *P < 0.01 in comparison to control group (repeated measures ANOVA and Tukey’s post hoc test)

Mephedrone at doses of 10 and 20 mg/kg significantly increased DA release in rat striatum and frontal cortex with maximum ca. 1 200 and 300 % of basal level, respectively, between 20 and 40 min after administration (Fig. 6). In nucleus accumbens, doses of 5, 10, and 20 mg/kg significantly increased the DA release to maximum 250 % of basal level at the highest dose (Fig. 6). Repeated measures ANOVA of these data showed a statistically significant effect of treatment groups [*F*_3,14_ = 76, *P* = 0 in striatum; *F*_3,14_ = 158, *P* = 0 in frontal cortex; *F*_3,12_ = 1601, *P* = 0 in nucleus accumbens], sampling period [*F*_8,112_ = 94, *P* = 0 in striatum; *F*_8,112_ = 86, *P* = 0 in frontal cortex; *F*_8,96_ = 96, *P* = 0 in nucleus accumbens], and the interaction between treatment groups and sampling period [*F*_24,112_ = 30, *P* = 0 in striatum; *F*_24,112_ = 16, *P* = 0 in frontal cortex; *F*_24,96_ = 44, *P* = 0 in nucleus accumbens].

**Fig. 7:**
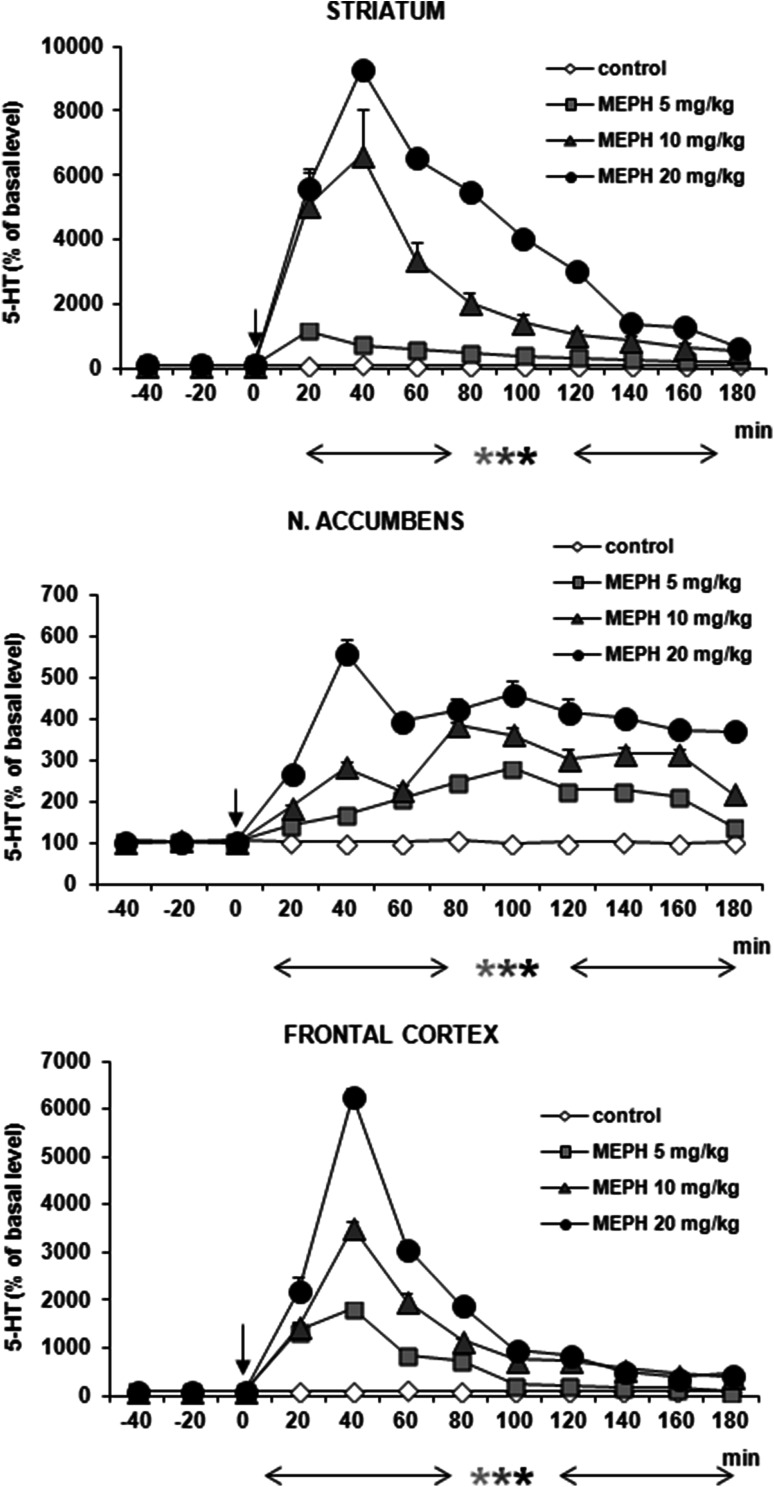
Effect of mephedrone (MEPH) on 5-HT release in rat striatum, nucleus accumbens, and frontal cortex. Data are mean ± SEM (*n* = 4–5). Drug administration is indicated with an arrow. Basal extracellular level of 5-HT (pg/10 μl) was 0.24 ± 0.05 (striatum); 0.12 ± 0.01 (n. accumbens); and 0.14 ± 0.01 (frontal cortex). *P < 0.01 in comparison to control group (repeated measures ANOVA and Tukey’s post hoc test)

The effect of mephedrone on 5-HT release was more potent than on DA release. Mephedrone in all doses (5, 10, and 20 mg/kg) significantly increased 5-HT release by ca. 500 % of basal level in nucleus accumbens, while at doses of 10 and 20 mg/kg by ca. 8000 and 6000 % of basal level in striatum and frontal cortex (Fig. 7). Repeated measures ANOVA of these data showed a statistically significant effect of treatment groups [*F*_3,14_ = 403, *P* = 0 in striatum; *F*_3,14_ = 857, *P* = 0 in frontal cortex; *F*_3,12_ = 2056, *P* = 0 in nucleus accumbens], sampling period [*F*_8,112_ = 472, *P* = 0 in striatum; *F*_8,112_ = 446, *P* = 0 in frontal cortex; *F*_8,96_ = 67, *P* = 0 in nucleus accumbens], and the interaction between treatment groups and sampling period [*F*_24,112_ = 154, *P* = 0 in striatum; *F*_24,112_ = 99, *P* = 0 in frontal cortex; *F*_24,96_ = 22, *P* = 0 in nucleus accumbens].

### DA and 5-HT Release in the Rat Striatum, Nucleus Accumbens, and Frontal Cortex After Administration of MDMA

**Fig. 8:**
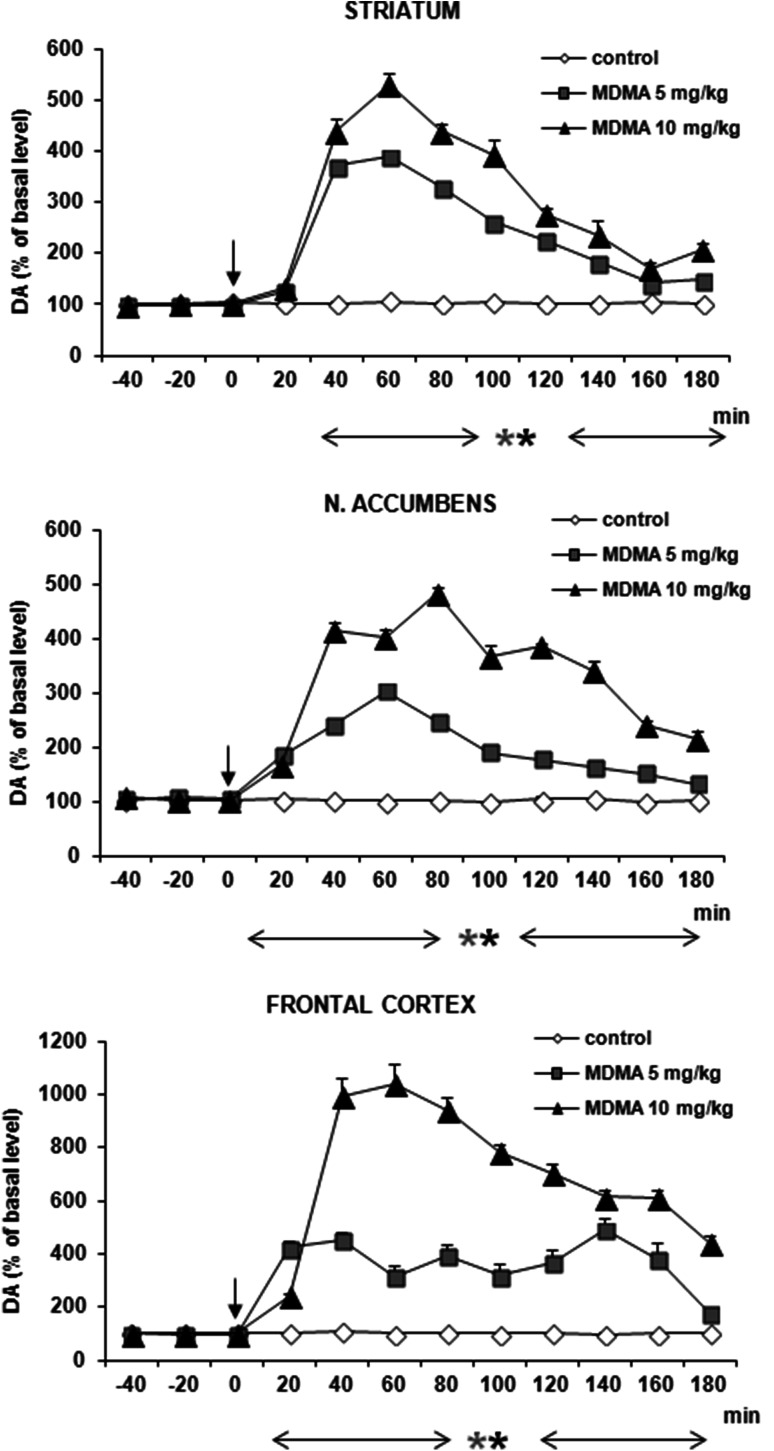
Effect of MDMA on DA release in rat striatum, nucleus accumbens, and frontal cortex. Data are mean ± SEM (*n* = 4–5). Drug administration is indicated with an arrow. Basal extracellular level of DA (pg/10 μl) was 18.7 ± 1.0 (striatum); 1.01 ± 0.14 (n. accumbens); and 1.02 ± 0.24 (frontal cortex). *P < 0.01 in comparison to control group (repeated measures ANOVA and Tukey’s post hoc test)

MDMA at doses of 5 and 10 mg/kg significantly increased DA release by ca. 500, 400, and 1000 % of basal level at 60–80 min after administration in striatum, nucleus accumbens and frontal cortex, respectively (Fig. 8). Repeated measures ANOVA of these data showed a statistically significant effect of treatment groups [*F*_2,9_ = 193, *P* = 0 in striatum; *F*_2,9_ = 1809, *P* = 0 in frontal cortex; *F*_2,9_ = 600, *P* = 0 in nucleus accumbens], sampling period [*F*_8,72_ = 169, *P* = 0 in striatum; *F*_8,72_ = 46, *P* = 0 in frontal cortex; *F*_8,72_ = 120, *P* = 0 in nucleus accumbens], and the interaction between treatment groups and sampling period [*F*_16,72_ = 48, *P* = 0 in striatum; *F*_16,72_ = 49, *P* = 0 in frontal cortex; *F*_16,72_ = 56, *P* = 0 in nucleus accumbens].

**Fig. 9:**
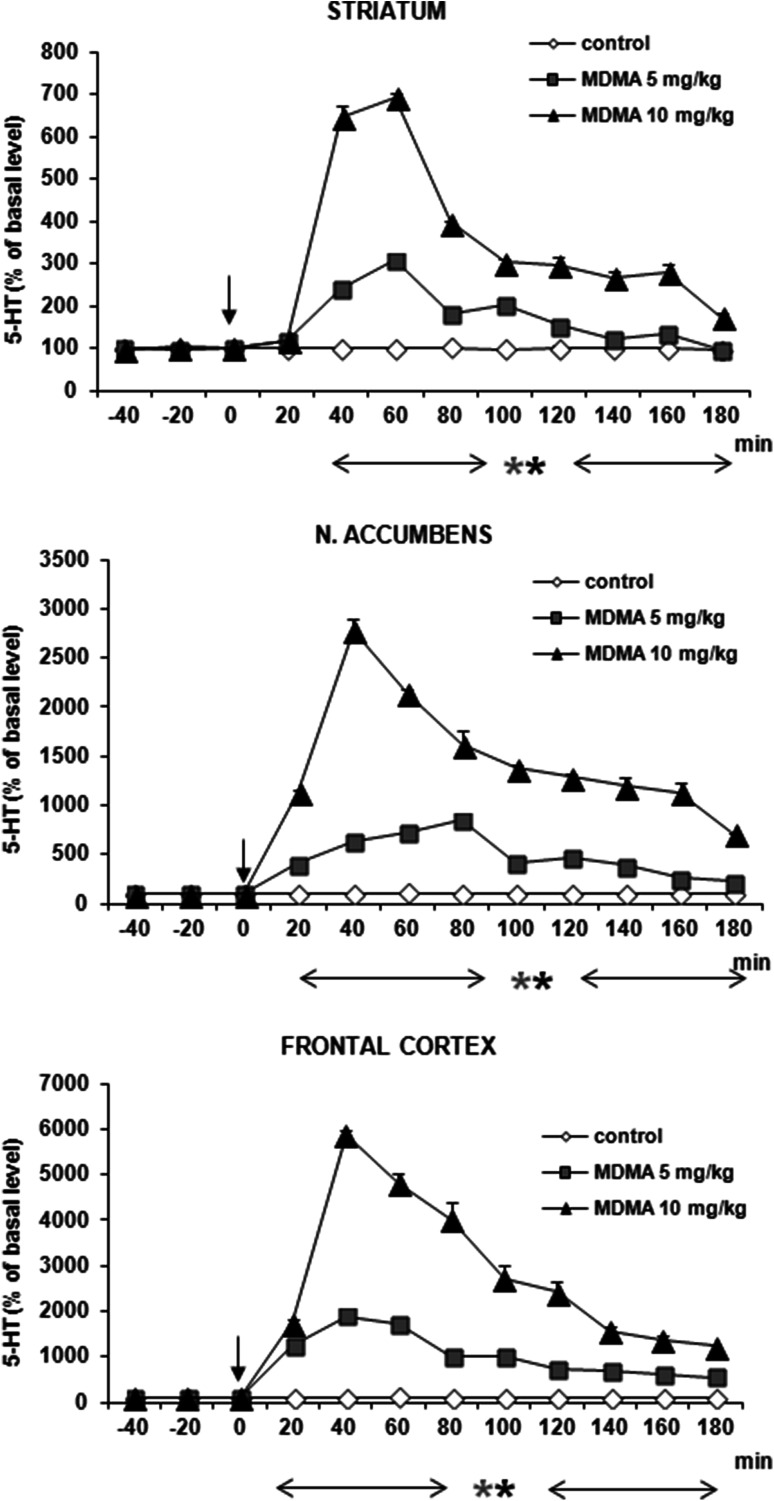
Effect of MDMA on 5-HT release in rat striatum, nucleus accumbens, and frontal cortex. Data are mean ± SEM (*n* = 4–5). Drug administration is indicated with an arrow. Basal extracellular level of 5-HT (pg/10 μl) was 0.51 ± 0.14 (striatum); 0.19 ± 0.04 (n. accumbens); and 0.22 ± 0.03 (frontal cortex). *P < 0.01 in comparison to control group (repeated measures ANOVA and Tukey’s post hoc test)

The effect of MDMA on 5-HT release was comparable in its potency to the effect on DA release in striatum reaching 600 % of basal level at 60 min after administration (Fig. 9). However, MDMA markedly increased 5-HT release by ca. 2500 and 6000 % of basal level in nucleus accumbens and frontal cortex (Fig. 9). Repeated measures ANOVA of these data showed a statistically significant effect of treatment groups [*F*_2,9_ = 523, *P* = 0 in striatum; *F*_2,9_ = 311, *P* = 0 in frontal cortex; *F*_2,9_ = 691, *P* = 0 in nucleus accumbens], sampling period [*F*_8,72_ = 303, *P* = 0 in striatum; *F*_8,72_ = 193, *P* = 0 in frontal cortex; *F*_8,72_ = 131, *P* = 0 in nucleus accumbens], and the interaction between treatment groups and sampling period [*F*_16,72_ = 136, *P* = 0 in striatum; *F*_16,72_ = 101, *P* = 0 in frontal cortex; *F*_16,72_ = 71, *P* = 0 in nucleus accumbens].

### Total Effect of PMA, PMMA, Mephedrone, and MDMA on DA and 5-HT Release in Brain Regions

The summarized time-course effect defined as an area under the curve (AUC) after administration of 10 mg/kg of each drug in all studied brain regions is provided in Table 1. PMA, PMMA, and mephedrone were nearly equally potent in increasing DA release in striatum. Mephedrone was slightly weaker in increasing DA release in nucleus accumbens and frontal cortex than PMA and PMMA, while MDMA remarkably increased DA release in all brain regions being the most potent in frontal cortex.

**Table 1:** Total effect of PMA, PMMA, mephedrone (MEPH), and MDMA at a dose of 10 mg/kg on DA and 5-HT release in the rat striatum, n. accumbens, and the frontal cortex

Treatment (mg/kg)	Striatum	N. accumbens	Frontal cortex
DA (AUC % of basal level) mean ± SEM			
Control	903 ± 11	910 ± 13	924 ± 13
PMA 10	2072 ± 80*	2237 ± 26*	2909 ± 76*
PMMA 10	2111 ± 104*	2307 ± 57*	2476 ± 32*
MEPH 10	1902 ± 112*	1593 ± 14*	1280 ± 49*
MDMA 10	2825 ± 109*	3009 ± 71*	6364 ± 109*
5-HT (AUC % of basal level) mean ± SEM			
Control	897 ± 16	898 ± 5	922 ± 7
PMA 10	11,170 ± 291*	3286 ± 54*	16,962 ± 740*
PMMA 10	3502 ± 144*	3002 ± 105*	4200 ± 38*
MEPH 10	21,642 ± 380*	2581 ± 19*	11,036 ± 222*
MDMA 10	3170 ± 81*	13,370 ± 419*	25,773 ± 1231*

Values are mean ± SEM (*n* = 4) and express area under the curve (AUC) of the percent of basal level. * *P* < 0.001 versus control group (one-way ANOVA and Tukey’s post hoc test)

PMA and mephedrone markedly enhanced 5-HT release in striatum, but the increase induced by mephedrone was ca. twofold stronger than that by PMA. PMMA and MDMA produced weaker and nearly equal effect on 5-HT release in this brain region. PMA, PMMA, and mephedrone induced comparable increase in 5-HT release in nucleus accumbens and were nearly threefold weaker than MDMA. However, PMMA was weakest in increasing 5-HT release in frontal cortex, as PMA, mephedrone, and MDMA enhanced 5-HT release by ca. fourfold, two-and-a-half-fold, and sixfold, respectively, when compared to PMMA (Table 1).

### The Tissue Content of DA, 5-HT, and Their Metabolites in the Rat Striatum, Nucleus Accumbens, and Frontal Cortex After Administration of PMA

PMA at doses 5 and 10 mg/kg significantly enhanced DA tissue content in nucleus accumbens (*P* < 0.01), at higher dose in frontal cortex (*P* < 0.01), and did not change its level in striatum (Table 2). The DOPAC tissue level was increased by both PMA doses in frontal cortex (*P* < 0.01), by higher dose in striatum (*P* < 0.05), and remained unchanged in nucleus accumbens (Table 2). PMA in both doses significantly enhanced HVA tissue level in frontal cortex (*P* < 0.01), while it did not change its level in striatum and nucleus accumbens (Table 2).

**Table 2:** Tissue content of DA, DOPAC, HVA, 5-HT, and 5-HIAA in rat striatum, nucleus accumbens, and frontal cortex measured 3 h after administration of PMA, PMMA, and mephedrone (MEPH); * *P* < 0.05, ** *P* < 0.01 versus control group (one-way ANOVA and Tukey’s post hoc test)

Treatment (mg/kg)	DA	DOPAC	HVA	5-HT	5-HIAA
Striatum pg/mg wt ± SEM (*n*)					
Control	10,606 ± 402 (9)	1188 ± 51 (9)	771 ± 37 (9)	698 ± 49 (7)	706 ± 50 (9)
PMA 5	11,705 ± 459 (5)	1078 ± 98 (5)	795 ± 56 (5)	646 ± 57 (5)	520 ± 56 (5)*
PMA 10	11,733 ± 329 (5)	855 ± 54 (5)*	755 ± 56 (5)	656 ± 41 (5)	493 ± 36 (5)*
PMMA 5	11,734 ± 281 (4)	1062 ± 28 (4)	811 ± 60 (4)	846 ± 75 (4)	608 ± 23 (4)
PMMA 10	16,092 ± 1846 (6)**	924 ± 28 (6)*	1300 ± 130 (6)**	1162 ± 121 (6)*	493 ± 19 (6)*
MEPH 10	13,349 ± 612 (5)	1249 ± 58 (5)	1053 ± 79 (5)*	762 ± 56 (5)	655 ± 36 (5)
MEPH 20	20,422 ± 1957 (5)**	1864 ± 162 (5)*	1485 ± 109 (5)**	715 ± 86 (5)	854 ± 94 (5)
Nucleus accumbens pg/mg wt ± SEM (*n*)					
Control	9992 ± 341 (12)	1560 ± 102 (8)	1137 ± 104 (10)	912 ± 64 (11)	759 ± 54 (12)
PMA 5	12,608 ± 1225 (5)**	1447 ± 124 (5)	1235 ± 106 (5)	1326 ± 71 (5)*	935 ± 89 (5)
PMA 10	18,117 ± 988 (5)**	1860 ± 145 (5)	1221 ± 128 (5)	1685 ± 212 (5)**	1285 ± 84 (5)**
PMMA 5	13,668 ± 781 (5)**	1560 ± 95 (4)	1129 ± 139 (5)	1139 ± 142 (4)	515 ± 74 (4)*
PMMA 10	17,076 ± 990 (5)**	1331 ± 73 (6)	1027 ± 94 (5)	1180 ± 124 (6)	465 ± 49 (6)*
MEPH 10	15,443 ± 934 (5)**	2113 ± 123 (5)**	1212 ± 147 (5)	877 ± 62 (5)	861 ± 37 (5)
MEPH 20	19,235 ± 757 (5)**	2608 ± 189 (5)**	1691 ± 67 (5)*	1045 ± 136 (5)	763 ± 54 (5)
Frontal cortex pg/mg wt ± SEM (*n*)					
Control	1163 ± 49 (9)	380 ± 47 (14)	127 ± 10 (11)	637 ± 49 (12)	318 ± 44 (10)
PMA 5	1247 ± 81 (5)	193 ± 21 (5)**	205 ± 15 (5)**	1291 ± 59 (5)**	567 ± 46 (5)*
PMA 10	1620 ± 102 (5)**	131 ± 8 (5)**	253 ± 19 (5)**	1501 ± 12 (5)**	870 ± 42 (5)**
PMMA 5	849 ± 31 (4)*	285 ± 54 (4)	150 ± 16 (4)	716 ± 34 (4)	226 ± 21 (4)
PMMA 10	638 ± 49 (6)**	190 ± 25 (6)**	294 ± 46 (6)**	1205 ± 71 (6)**	279 ± 21 (6)
MEPH 10	526 ± 64 (5)**	174 ± 34 (5)**	85 ± 1.5 (5)*	423 ± 46 (5)*	291 ± 81 (5)
MEPH 20	380 ± 65 (5)**	86 ± 5 (5)**	70 ± 3.1 (5)**	332 ± 7.8 (5)**	288 ± 35 (5)*

5-HT tissue level was potently increased by both doses of PMA in frontal cortex (*P* < 0.01) as it did in nucleus accumbens at doses of 5 and 10 mg/kg (*P* < 0.05 and *P* < 0.01, respectively), but remained unchanged in striatum (Table 2). 5-HIAA content was decreased by both PMA doses in striatum (*P* < 0.05), but it was increased by PMA (10 mg/kg, *P* < 0.01) in nucleus accumbens and in frontal cortex by 5 and 10 mg/kg of PMA (*P* < 0.05 and *P* < 0.01, respectively).

### The Tissue Content of DA, 5-HT, and Their Metabolites in the Rat Striatum, Nucleus Accumbens, and Frontal Cortex After Administration of PMMA

PMMA at a dose of 10 mg/kg significantly increased DA content in striatum (*P* < 0.01) and at both doses in nucleus accumbens (*P* < 0.01), but decreased its tissue level at doses of 5 and 10 mg/kg (*P* < 0.05 and *P* < 0.01, respectively) in frontal cortex (Table 2). Higher doses of PMMA significantly decreased DOPAC tissue level in striatum (*P* < 0.05) and in frontal cortex (*P* < 0.01) but did not change its level in nucleus accumbens (Table 2). HVA tissue level was increased by PMMA (10 mg/kg) in striatum and frontal cortex (*P* < 0.01) but remained unchanged in the nucleus accumbens (Table 2).

5-HT content was increased by PMMA at a dose of 10 mg/kg in striatum (*P* < 0.05) and in frontal cortex (*P* < 0.01), while it was not affected by PMMA in nucleus accumbens (Table 2). PMMA in both doses decreased 5-HIAA tissue level in nucleus accumbens (*P* < 0.05) and at a higher dose in the striatum (*P* < 0.05) but did not influence 5-HIAA content in frontal cortex (Table 2).

### The Tissue Content of DA, 5-HT and Their Metabolites in the Rat Striatum, Nucleus Accumbens and Frontal Cortex After Administration of Mephedrone

Mephedrone studied in doses of 10 and 20 mg/kg enhanced DA content in frontal cortex (*P* < 0.01) and nucleus accumbens, while in striatum only at a higher dose (Table 2). Similarly, DOPAC level was significantly increased by both mephedrone doses in frontal cortex and nucleus accumbens (*P* < 0.01), while that in striatum by the higher dose only (*P* < 0.05). HVA tissue level was significantly increased in striatum and frontal cortex by 10 and 20 mg/kg of mephedrone (*P* < 0.05 and *P* < 0.01, respectively), while in nucleus accumbens, its level was enhanced by the higher dose only (*P* < 0.05).

5-HT and 5-HIAA tissue levels were not changed by mephedrone at both doses tested in striatum and nucleus accumbens (Table 2). In frontal cortex, mephedrone significantly decreased (at both doses) 5-HT tissue level (*P* < 0.01), while 5-HIAA level was significantly decreased by a higher dose only (*P* < 0.05).

## Discussion

The main finding of our study is that although some differences in potency were observed, mephedrone, PMA, and PMMA markedly increased DA and 5-HT release in the rat striatum, the nucleus accumbens, and the frontal cortex and induced changes in the monoamine turnover in the rat brain.

In our study, single doses of PMA increased DA release in rat striatum and nucleus accumbens with similar potency but with a slightly greater effect in the frontal cortex. However, PMA effect on 5-HT was stronger than on DA release in all brain regions. These data are consistent with in vitro monoamine uptake inhibition profile of PMA since it was nearly 30-fold less potent at the DAT versus SERT (Simmler et al. 2014). Our results are in line with study of Gough et al. (2002) who reported an increase in striatal DA and 5-HT in freely moving rats, but at much higher dose regimen (10 and 20 mg/kg). The difference in potency of the drug may be due to rat strain or housing conditions, such as room temperature and single versus grouped housing, which strongly influence the effect of psychostimulant drugs (Green and Nutt 2014). Our results are also consistent with those of Matsumoto et al. (2014) who found remarkable increase in the level of 5-HT in rat hippocampus but lower increase in striatal DA level after PMA at a dose of 5 mg/kg. Callaghan et al. (2005) suggest in chronoamperometric studies that the more prominent PMA effect on 5-HT level observed in rat striatum may be related with its clearance by both the SERT and DAT.

Increase in DA content in nucleus accumbens and frontal cortex, with concomitant decrease or no change in DOPAC or HVA content in those brain regions, seems to result from DAT inhibition and disturbed intracellular monoamine metabolism via MAO, caused by PMA (Matsumoto et al. 2014). Similarly, DAT transport inhibition seems to be responsible for the decrease in striatal DOPAC content caused by the high dose of PMA (Gough et al. 2002). Serotonin neurons in nucleus accumbens and frontal cortex are also markedly affected by PMA. Increase in 5-HT and its metabolite speaks for stimulatory effect of PMA on 5-HT synthesis. It is possible that PMA besides promoting 5-HT release may have direct impact on serotonin neurons via stimulation of 5-HT2A receptors (Simmler et al. 2014). This effect seems to be region specific as it is not observed in striatum where the decrease in 5-HT metabolism may result from SERT inhibition by PMA (Callaghan et al. 2005; Daws et al. 2000; Freezer et al. 2005).

PMMA, similarly to PMA, is a potent SERT but weak DAT inhibitor (Simmler et al. 2014). In our work, it increased DA and 5-HT release in all studied brain regions, but PMMA effect on 5-HT release in striatum and frontal cortex was not as strong as that of PMA; nevertheless, it was greater than on DA release in all tested regions. These data are in agreement with results reported by Matsumoto et al. (2014) who observed remarkable increase in hippocampal level of 5-HT by PMMA but a slight increase in levels of DA in striatum. Interestingly, in our study, the time-course effect of PMMA on DA release varied between brain regions. The maximal increase in DA level was at 80 min in striatum and 40 min in frontal cortex but was delayed in nucleus accumbens to 160 min after drug administration. Similar pattern as for DA was also observed in 5-HT release with similar effect in magnitude in striatum and frontal cortex and more extended time course in nucleus accumbens. It seems that increase in cortical DA or 5-HT level may account for the delay in accumbal DA release via GABA-ergic cortical projections to the nucleus accumbens (Lee et al. 2014). Moreover, formation of PMA as an active N-demethylated metabolite of PMMA may relate to delayed effect on DA and 5-HT release observed in the nucleus accumbens but not in other brain regions (Rohanova and Balikova 2009).

PMMA, similarly to PMA also enhanced DA content in striatum and nucleus accumbens. However, its influence on DOPAC and HVA level in both regions was not quite consistent. Similarly to mephedrone, PMMA decreased DA and DOPAC content in frontal cortex, which suggests regulation of DA cortical neurons by released 5-HT or directly by PMMA via 5HT2A/2C receptors (Bankson and Yamamoto 2004; Di Giovanni et al. 2001; Di Matteo et al. 2001; Gudelsky and Yamamoto 2008). Increase in striatum and frontal cortex or lack of change in nucleus accumbens of 5-HT content with concomitant decrease or no change in 5-HIAA content seems to reflect the effect of PMMA on SERT inhibition, which may lead to malfunction of 5-HT metabolism.

Mephedrone, as a beta-keto-analog of amphetamines, is a nonselective substrate for plasma membrane monoamine transporters that also releases monoamines similarly to classic amphetamines (Baumann et al. 2012; Pifl et al. 2015; Simmler et al. 2014). In our study, mephedrone stimulated DA and 5-HT release in striatum, nucleus accumbens, and frontal cortex, with the magnitude of effect on 5-HT being greater. These findings are consistent with previous studies that report relatively stronger in vitro effect of mephedrone on 5-HT release than on DA release in HEK 293 cells and lower DAT: SERT ratio (Rickli et al. 2015). Our data resemble those of Kehr et al. (2011), who showed rapid and significant increase in 5-HT and DA levels in nucleus accumbens of awake rats after subcutaneous administration of 1 and 3 mg/kg of mephedrone. Similarly to our results, these researchers observed that mephedrone was a more preferential releaser of 5-HT than DA. In recent study of Shortall et al. (2015), three injections of mephedrone (10 mg/kg) in 2-h intervals increased striatal release of DA and 5-HT following each injection. The magnitude of DA increase was similar to that shown in our work, but we observed a more potent increase in striatal 5-HT level after single administration of mephedrone. The difference in neuronal response to mephedrone in study of Shortall et al. (2015) and ours may be related with different rat strains used in both studies as well as technical details such as one drug injection versus three injections. The increase in DA release, which was short in duration in striatum or frontal cortex and a more prolonged in nucleus accumbens, corresponds with locomotor stimulation and self-administration reported by Motbey et al. (2014). What is more, those observed changes in DA release point out the addiction liability of mephedrone. As a consequence of predominant increase in 5-HT release and marked direct affinity to 5-HT2A receptor (Rickli et al. 2015) mephedrone may produce hallucinogenic effect (Nicholas 2004). By stimulation of DA release from mesolimbic and mesocortical dopamine terminals indirectly via DAT inhibition or directly via 5-HT2A receptors in the ventral tegmental area (VTA), mephedrone may evoke addiction and increase locomotor activity (Green et al. 2014; Iversen et al. 2014).

In our study, single doses of mephedrone caused marked increase in striatal and accumbal DA tissue content as well as its metabolites, DOPAC and HVA. This effect indicates an increase in DA synthesis mediated possibly postsynaptically via D1/D2 dopamine receptors situated in medium spiny GABA-ergic neurons or through 5-HT2A receptors activation (by released 5-HT), which are located on glutamatergic neurons, projecting from striatum or nucleus accumbens to nigral or VTA regions (Di Matteo et al. 2008). In contrast, mephedrone decreased the content of DA, DOPAC, and HVA in the frontal cortex. This indicates reductions in DA synthesis which may be mediated indirectly by released 5-HT. As mesolimbic and mesocortical neurons are highly regulated by inhibitory GABA-ergic neurons, their stimulation by released 5-HT via 5-HT2C receptors may inhibit VTA neurons and lead to a decrease in DA synthesis (Bankson and Yamamoto 2004; Di Giovanni et al. 2001; Di Matteo et al. 2001). Interestingly, while mephedrone did not affect serotonin turnover in striatum and nucleus accumbens, it decreased 5-HT and 5-HIAA contents in the frontal cortex, suggesting the possible acute damaging properties of mephedrone. This observation is interesting in terms of neurotoxicity of the drug, previously noted only after “binge” model of administration in rats and mice (Angoa-Pérez et al. 2013; Baumann et al. 2012; López-Arnau et al. 2015; Hadlock et al. 2011).

In comparison to PMA, PMMA, and mephedrone, MDMA (used here as a reference drug) produced a similar effect on DA release but markedly promoted the release of 5-HT in nucleus accumbens and frontal cortex. Similarly to the *para*-methoxyamphetamines and mephedrone, MDMA has a higher affinity for SERT than DAT and is more potent in releasing 5-HT than DA (Rothman and Baumann 2003). The effect of equal doses of PMMA, mephedrone, and MDMA on DA and 5-HT release presented in Table 1 indicates apparent differences between tested drugs. The amount of DA released in striatum was similar for PMA, PMMA, and mephedrone; however, mephedrone was less potent in nucleus accumbens and frontal cortex. The action of MDMA on DA release was more robust than PMA, PMMA, and mephedrone in all brain regions. The ability of PMA and mephedrone to increase 5-HT release was more prominent than PMMA and MDMA in striatum. The effect of PMA, mephedrone, and MDMA on 5-HT release was stronger than PMMA in the frontal cortex. PMA, PMMA and mephedrone were equally efficacious in nucleus accumbens, while MDMA produced a much stronger effect in this brain region. These data are in agreement with the studies of Matsumoto et al. (2014) who found that PMA, PMMA, and MDMA have a weaker effect on DA than that on 5-HT release.

Augmentation of DA and 5-HT release by new psychoactive drugs may be an indication of neurotoxicity observed after prolonged treatment—as evidenced in numerous studies for MDMA, the basic substance for development of new structural congeners (Capela et al. 2009; Molliver et al. 1990). However, there is only one study showing long-term (possibly neurotoxic) effects of high repeated doses (80 mg/kg for 4 days) of PMA and PMMA on serotonin neurons in hippocampus and frontal cortex (Steele et al. 1992). In another study, a 4-day administration of PMA at doses 10–20 mg/kg did not reduce cortical and hippocampal 5-HT content in spite of a decrease in 5-HIAA and SERT density (Callahan et al. 2006, 2007). Nevertheless, repeated treatment with new psychoactive drugs may cause disturbance in DA compartmentation by amphetamine derivatives which further leads to DA autooxidation and generation of oxidative stress (Barbosa et al. 2012; Halliwell 2006). Similarly, potent increase in 5-HT extracellular level may give rise to formation of dihydroxybitryptamine oxidative products such as tryptamine-4,5-diones (Ximenes et al. 2009). Those detrimental processes may cause retrograde degeneration of 5-HT terminals observed after long-term administration of psychostimulants (Granado et al. 2011). Moreover, potent increase in 5-HT extracellular level, particularly in frontal cortex, points to hallucinogenic properties of mephedrone and amphetamine derivatives as it was shown for mescaline (Aghajanian and Marek 1999; González-Maeso and Sealfon 2009). The neurochemical basis of this effect is stimulation of 5-HT2A receptors situated in pyramidal cells in cortex and release of glutamate. In consequence, activation of NMDA and AMPA receptors leads to further stimulation of excitatory pathways projecting to VTA and nucleus raphe region and their activation. This mechanism seems to be responsible for hallucinogenic properties and abuse potential of cathinones and amphetamine derivatives (Capela et al. 2009; Creehan et al. 2015; Iversen et al. 2014; Nicholas 2004).

To summarize, the novel psychoactive drugs have the ability to markedly influence DA and 5-HT release in rat brain and to produce changes in monoamine synthesis and metabolism. Depending on the brain region, mephedrone, PMA, and PMMA show different pharmacological profiles and potency in their action. Observed changes in monoamine release and turnover suggest hallucinogenic, abuse, and neurotoxic risk of these drugs (See diagram below). Further studies with repeated treatment are necessary to understand the mechanism underlying the dopaminergic and serotoninergic neurotoxicity produced by those drugs.



Diagram shows graphical presentation of mephedrone, PMA, and PMMA mechanism leading to hallucinogenic effects, abuse, and neurotoxicity.
